# Cold atmospheric plasma treatment enhances recombinant model protein production in yeast *Pichia pastoris*

**DOI:** 10.1038/s41598-023-34078-y

**Published:** 2023-04-26

**Authors:** Zeinab Kabarkouhi, Sareh Arjmand, Seyed Omid Ranaei Siadat, Babak Shokri

**Affiliations:** 1 Laser and Plasma Research Institute, Shahid Beheshti University, P.O. Box: 1983969411, Tehran, Iran; 2 Protein Research Center, Shahid Beheshti University, P.O. Box: 1983969411, Tehran, Iran

**Keywords:** Plasma physics, Biochemistry, Biotechnology, Molecular biology

## Abstract

Cold atmospheric pressure plasma (CAP) has been described as a novel technology with expanding applications in biomedicine and biotechnology. In the present study, we provide a mildly stressful condition using non-lethal doses of CAP (120, 180, and 240 s) and evaluate its potential benefits on the recombinant production of a model protein (enhanced green fluorescent protein (eGFP)) in yeast *Pichia pastoris*. The measured eGFP fluorescence augmented proportional to CAP exposure time. After 240 s treatment with CAP, the measured fluorescent intensity of culture supernatant (after 72 h) and results of real-time PCR (after 24 h) indicated an 84% and 76% increase in activity and related RNA concentration, respectively. Real-time analysis of a list of genes involved in oxidative stress response revealed a significant and durable improvement in their expression at five h and 24 h following CAP exposure. The improvement of the recombinant model protein production may be partly explained by the impact of the RONS on cellular constituents and altering the expression of specific stress genes. In conclusion, using CAP strategy may be considered a valuable strategy to improve recombinant protein production, and deciphering the molecular background mechanism could be inspiring in the reverse metabolic engineering of host cells.

## Introduction

*Pichia pastoris* (*syn. Komagataella phaffii*) yeast serves as a great host for recombinant protein production and has extensively been used for pharmaceuticals and industrial protein manufacturing^1^. This yeast provides fascinating advantages as the host, including easy manipulation and cost-effective and easy large-scale production processes^2,3^. It can be planned to utilize different promoters and appropriate carbon sources for growth and recombinant protein production in *Pichia pastoris*. Amongst them, using the strong alcohol oxidase 1 promoter (pAOX1), induced by low methanol concentration, is highly interesting in the industrial-scale production of recombinant proteins^4^.

After its development in 1970, recombinant protein production technology revolutionized different world industries, mainly the medicine and healthcare industry^5^. The rising prevalence of chronic diseases and the increasing desire for the replacement of chemical processes with biological ones, due to the demand for environmental considerations, led to significant global growth in the recombinant protein industry^6^. Growing global demand for recombinant protein production and the importance of its market necessitate improving the cost-effectiveness of recombinant protein manufacturing processes^7^. Different solutions (*e.g.,* increasing gene dosage, metabolic engineering, culture modification, optimization of environmental or bioreactor operational conditions) have been developed to effectively ameliorate the recombinant production yield and attain cost reduction^8^.

The cell redox status is one of the key factors in regulating cellular physiology that significantly modifies global gene expression profiling^9^. Oxidative stress can deviate the cellular redox homeostasis status and affect the cell survival of the fittest. The cells' response to stress is determined by the amount of stress administered. While the low levels of reactive oxygen species (ROS) and reactive nitrogen species (RNS) may function as signals for cell growth and survival, the higher levels may lead to cell cycle arrest or mass cell death^9,10^. The cells adjust to moderate stress by altering the synthesis of their stress proteins.

It has been shown that altering the expression of specific stress proteins can boost the recombinant protein production yield^11^. Co-expression of genes involved in the oxidative stress response could provide antioxidant defense and have exhibited promising helper factor effects on the recombinant protein yields in different hosts, including *Pichia pastoris*^12,13^.

Nowadays, inducing ROS in biological systems has found considerable medical and industrial applications^14^. ROS could be produced through chemical and physical processes^15^. Besides these conventional methods, plasma-based approaches have recently received much attention for their ability to generate substantial amounts of ROS^16^.

Plasma, considered the fourth state of matter, creates a cocktail of chemically reactive oxygen/nitrogen species (RONS), thermal impression, UV/visible emissions, and electromagnetic fields. RONS is believed to make a meaningful contribution to plasma-induced events. On the basis of the equilibrium between electrons, ions, and neutral species, plasma is categorized as either thermal plasma or non-thermal plasma (cold atmospheric pressure plasma (CAP)). Unlike thermal plasma, the innovative CAP technology is a plasma that is not in thermodynamic equilibrium, resulting in temperature disparities between plasma species; whereas the plasma electron temperature may reach tens of thousands of Kelvin, the temperature of the neutral gas is around room temperature. In addition, CAP devices, including plasma jet and dielectric barrier discharge (DBD) plasma, typically operate at room temperature, making them ideal for life science research and medical applications^17–19^.

Here, we investigate the effect of a laboratory-designed alternating current (AC) CAP jet on the recombinant production of enhanced green fluorescent protein (eGFP) as a model protein in yeast *Pichia pastoris*. The optimum treatment duration, RONS production and stability analysis, and evaluation of some RONS-responsive genes in mRNA levels have been considered for production optimization.

## Results

### Optical emission spectrometry (OES) and plasma parameters

**Figure 1:**
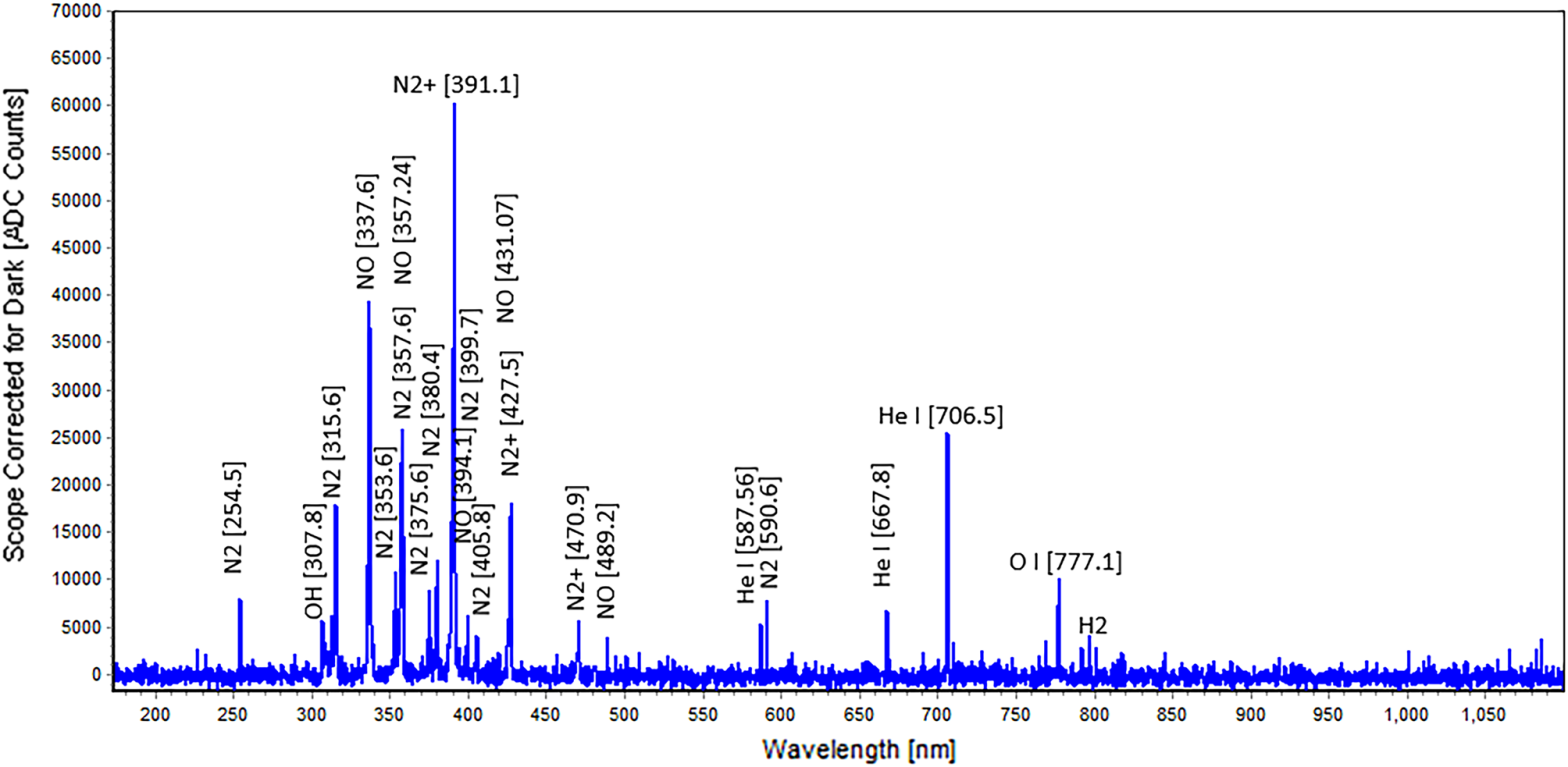
OES spectrum of He plasma.

Reactive species produced by plasma were defined from the OES of the used He plasma (Fig. 1). Atmospheric plasma emission shows the molecular bands of OH, N_2_, N_2_^+^, O, and ionized He. Molecular nitrogen (N_2_) and its ionized derivative (N_2_^+^) are dominant species.

**Figure 2:**
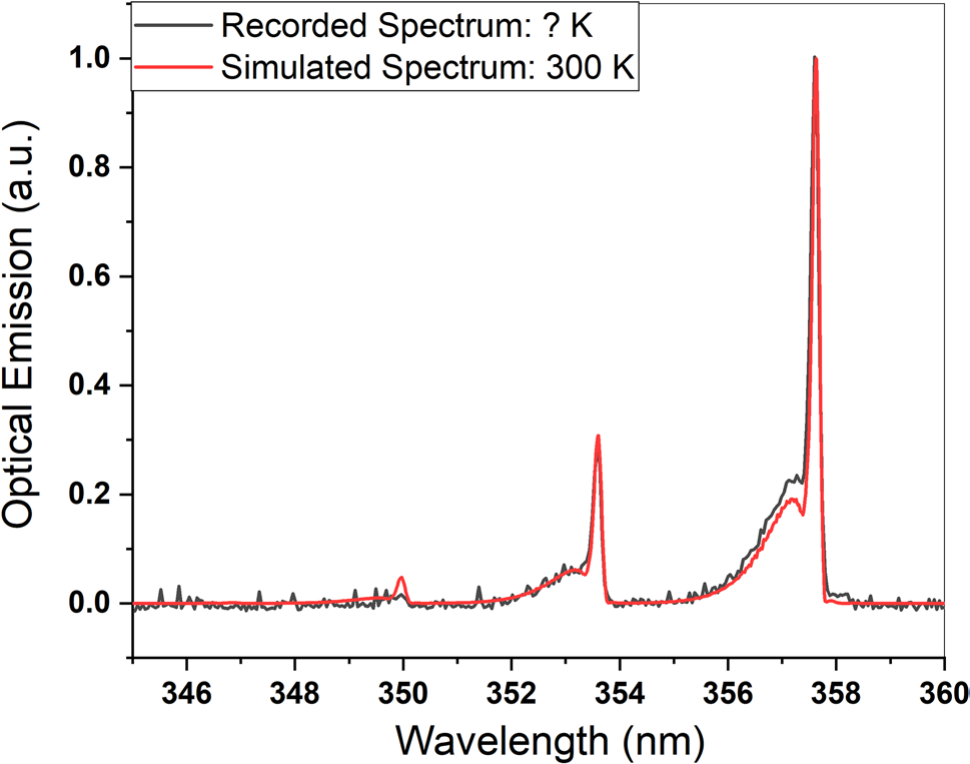
Specair simulated vs. experimental recorded spectrum.

The experimental calibrated and Specair simulated spectrum are displayed in Fig. 2. The simulated spectrum for rotational temperature (300 Kelvin (K) ≈ 27 °C), certifies it as cold plasma, which is known as a safe plasma jet for cell treatment.

### Effect of CAP on yeast cell growth and survival and protein production

**Figure 3:**
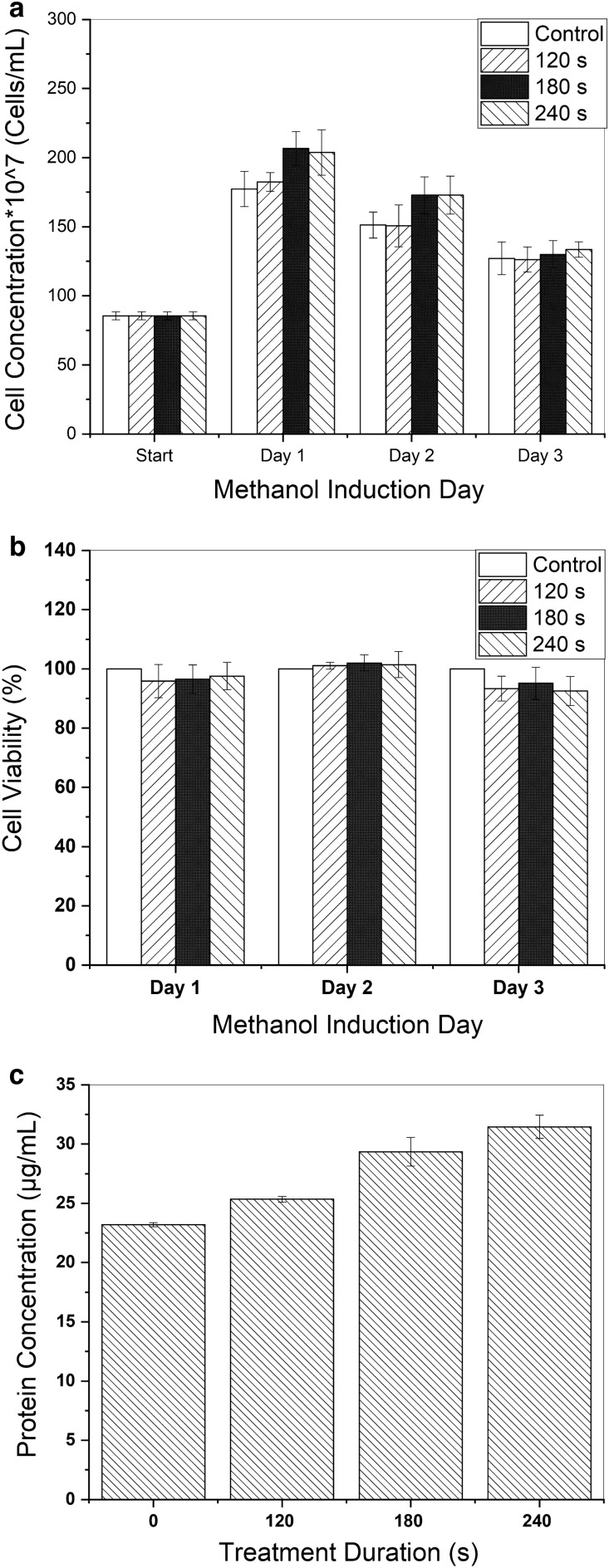
Effect of CAP treatment over three times duration on (**a**) Cell growth and (**b**) viability. The cell concentrations were obtained by measuring OD600 of the culture medium, and cell viability was assessed by MTT assay. (**c**) Total protein concentration on the last day of methanol induction, obtained by Bradford assay, indicated an increasing trend proportional to the treatment time. The error bars are standard deviations.

180 and 240 s CAP treatment led to a marginal increase (though not statically significant) in cell growth for two consecutive days, but this effect vanished on the third day following induction. For 120 s CAP exposure, no effect on cell growth was detected (Fig. 3a). The percentage of cell viability was calculated compared to non-treated cells (assumed as 100% alive). As depicted in Fig. 3b, the CAP-exposed cells displayed similar viability to the treated cells in three days of methanol induction. Despite the lack of significant changes in cell growth and viability, comparing the total protein concentration on the last day of methanol induction showed a considerable increase due to CAP treatment. The results of the Bradford assay indicated a 10, 27, and 36% increase in the cell culture total protein for 120, 180, and 240 s of CAP treatment, respectively, compared to the control (Fig. 3c).

### Fluorescence analysis

**Figure 4:**
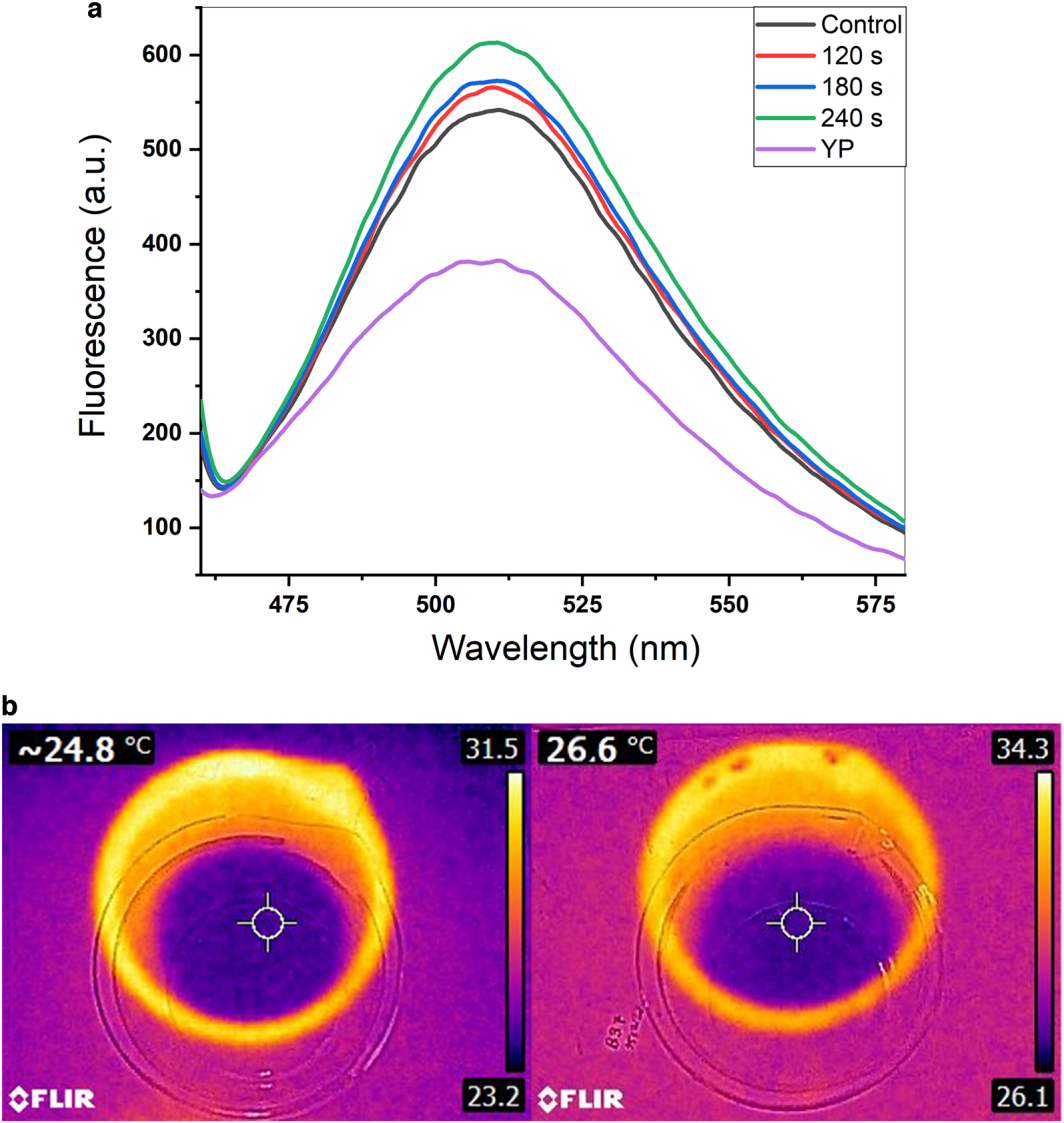
(**a**) The results of CAP treatment over various time periods on measured eGFP fluorescence activity. (**b**) Heat profile before (left) and after (right) 240 s CAP treatment.

The results of spectrofluorimetry are shown in Fig. 4a. The figure implies that as the CAP exposure time increased, the intensity of measured fluorescence in the cell culture medium was considerably amplified. For 120, 180, and 240 s exposure times, the fluorescence increase equals 45%, 53%, and 84%, respectively. The YP media has intrinsic fluorescence that was subtracted from the measured fluorescence. The fluorescence plot of the non-recombinant control supernatant was utterly consistent with the YP, so it is not shown in the figure.

### Analysis of pH and temperatures after CAP treatment

The results of heat photography indicated a maximally 1.8 ± 0.1 °C increase in temperature of the cell culture medium after 240 s of CAP treatment, which is not considered harmful to yeast cells (Fig. 4b). No changes in pH were detected for any of the CAP time exposures.

### Analysis of cultural media and intracellular RONS

**Figure 5:**
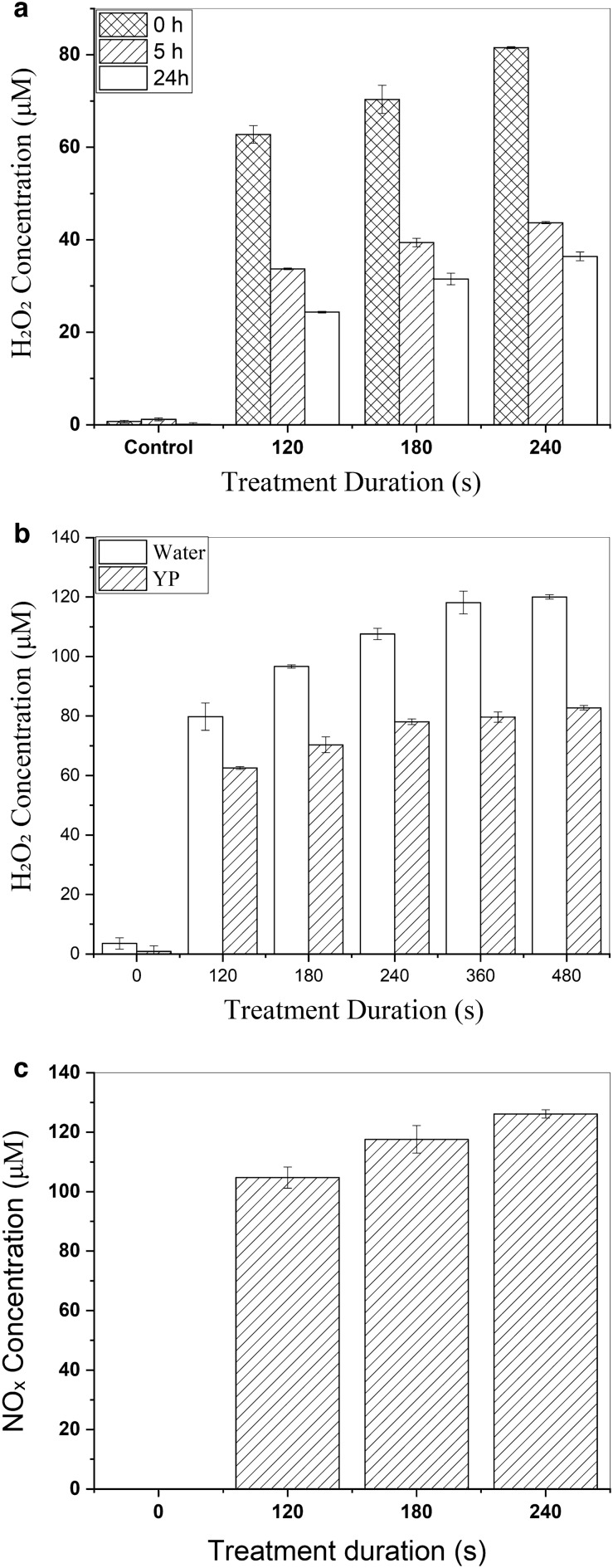
(**a**) Concentration and stability of produced H2O2 in culture media after CAP exposure. (**b**) Concentration of produced H_2_O_2_ in YP and distilled water. (**c**) Concentration of produced NO_x_ immediately after CAP exposure. Within five h no more NOx was detectable in the media. The error bars are standard deviations.

Immediately after CAP exposure, significant amounts of H_2_O_2_ are detectable in the culture media, which correlates linearly with exposure time duration. About 40% of this stable species was still detectable after 24 h of CAP exposure (Fig. 5a). To determine the medium's impact on H_2_O_2_ production, identical CAP exposure times and two additional longer time periods (360 and 480 s) were applied to YP and distilled water media (Fig. 5b). The higher amounts of H_2_O_2_ in distilled water could be attributed to the RONS scavenging role of YP medium ingredients.

Investigation of the production and stability of NO_x_ indicated that NO species are created at substantial levels and proportional to the exposure length right after the CAP treatment (Fig. 5c). However, these species had a short half-life; within five h, no NO_x_ was detectable.

**Figure 6:**
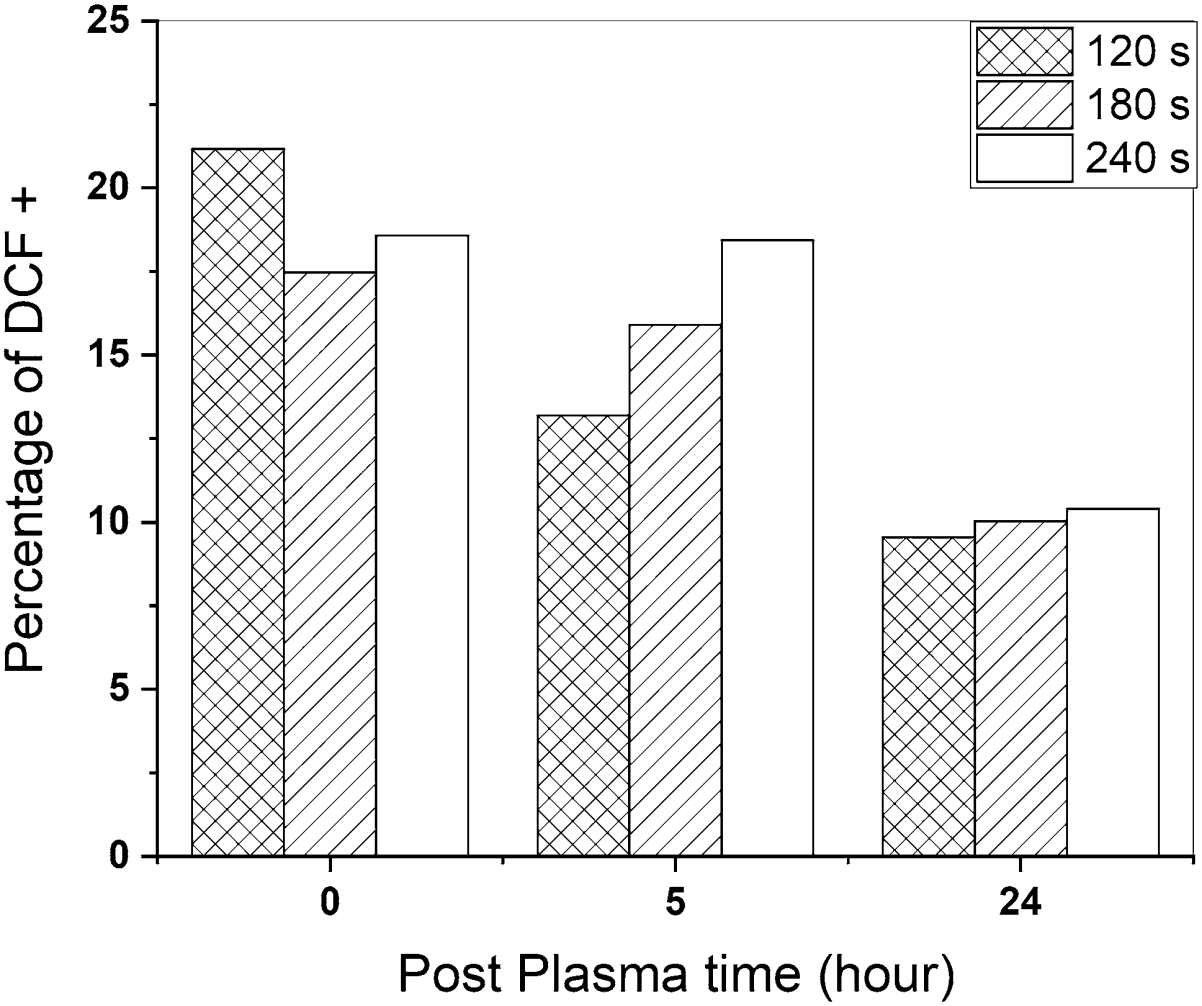
Intracellular RONS produced after CAP treatment and quantified by the H2DCF-DA indicator.

The proportion of DCF-labeled cells, in the analysis of intracellular RONS, normalized to the non-CAP treated cells, shows a significant increase after CAP treatment, which was remarkably stable within 24 h (Fig. 6). Contrary to the previous finding, 120 s of CAP exposure led to higher intracellular RONS; however, after 24 h, the differences in intracellular RONS related to exposure time disappeared.

### Gene expression analysis

**Figure 7:**
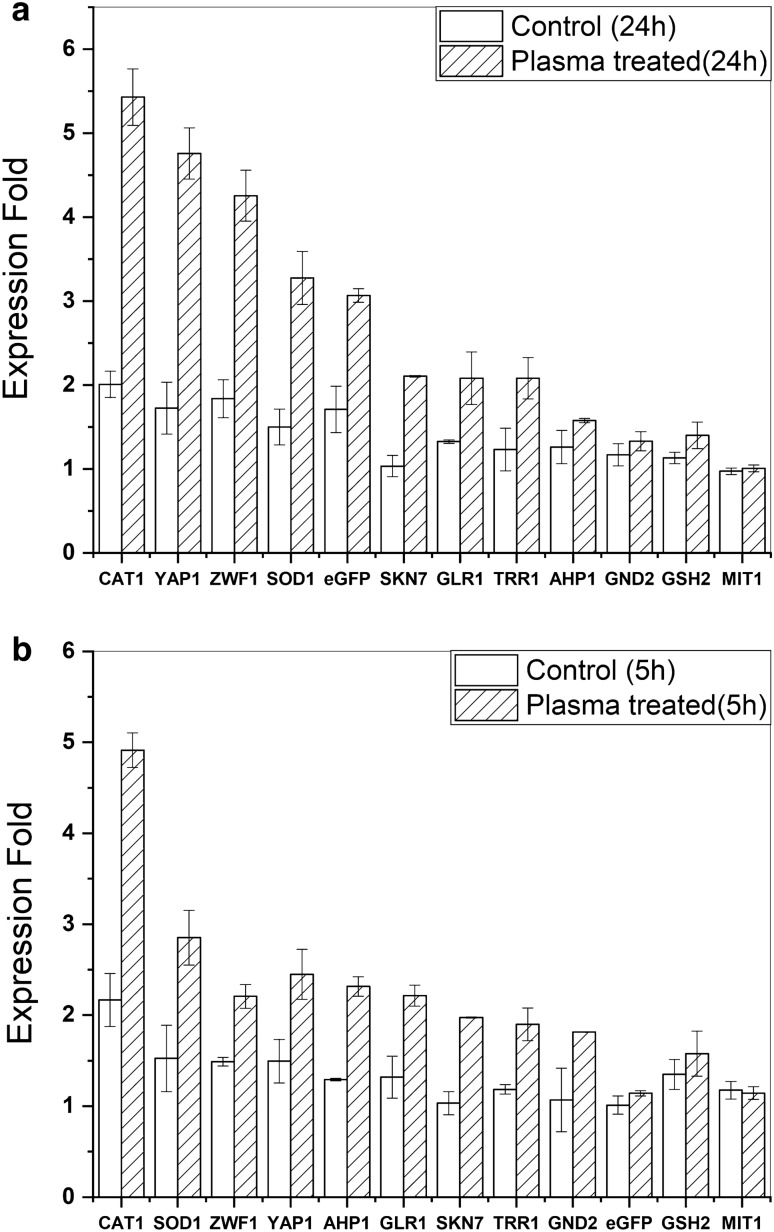
mRNA expression was analyzed after a. 5 h, b. 24 h after plasma treatment. The error bars are standard deviations.

The expression of 10 genes related to the antioxidant defense and transcription factors that responded to oxidative stress in *Pichia pastoris*, as well as Mit1 from the methanol metabolism pathway and the target gene (eGFP), has been analyzed 5 and 24 h following 240 s of CAP treatment. All selected genes had a statistically significant increase, which persisted or even enhanced after 24 h. The CAT1 expression showed an approximately 2.5-fold increase after 5 and 24 h of CAP treatment. YAP1, ZWF1, and SOD expression significantly increased after 24 h compared to the five h, while the increasing trend of AHP1, GND2, and GLR1 expression decreased. The target gene that has a similar expression in control and treated groups after 5 h, showed a 76% increment after 24 h. The Mit1 showed no changes in the effect of CAP treatment (Fig. 7a,b).

## Discussion

Recent years have seen an increased interest in the different biological and medical applications of ground-breaking CAP technology due to its efficacy and safety^20^. This technology impacts biological systems mainly by affecting different aspects of cellular physiology. Following cell treatment, CAP initiates a series of reactions such as membrane and morphology perturbations, cell metabolism changes, DNA breaks formation, and organelle modifications^21–23^.

CAPJs are popular plasma configurations in plasma medicine applications; a jet ionizes a guided stream of working gas in a cavity using two electrodes separated by a dielectric^24^. The plasma effluent is driven into the ambient air to interact with its oxygen and nitrogen molecules.

Increasing the capacity of microorganism hosts, whether eukaryotic or bacterial, to express heterologous proteins is always a goal deserving of a thorough investigation. Our earlier study proposed using a He plasma jet, performed by a pulsed DC power supply, to increase yeast *Pichia pastoris* productivity for the first time. Exposing the cells to CAP led to a considerable increase in the concentration of recombinant protein (phytase enzyme) as well as enzyme activity^25^. In the present study, we used the non-lethal doses of a newly constructed He plasma jet on a recombinant yeast cell, *Pichia pastoris,* expressing eGFP.

This new plasma jet is based on the same DBD arrangement as the one before, but unlike the one before, which was powered by a pulsed DC power supply, this setup uses the AC voltage. The pulsed voltage prevents heating of the target environment at high voltage (11 kV). Generally, the AC power supply creates less current and poses a lower risk of cell damage compared to the pulsed DC configuration^26^. Furthermore, the DC setup's non-optimized performance may occur due to charge buildup on the dielectric surface; however, the AC setup's polarity change is thought to provide a fix for this issue^27^. As shown in Fig. 4b, the 4 kV AC voltage used in this study also produced no thermal effects. Our findings, along with those of numerous other studies, demonstrated that CAP mainly affects cells by generating RONS, which can upset the cell intracellular redox homeostasis. Plasma induces RONS with different lifespans; the short-lived species transiently interact with cells, biomolecules, and their surrounding liquid environment and consequently form long-lived species called intermediates^28^.

Depending on the investigated cell type, CAP characteristics, and exposure intensity, the perturbation of redox balance can turn on unspecific targets that lead to cell death (oxidative distress) or induce specific targets and stimulate cellular processes (oxidative eustress); a phenomenon called hormesis model^29–31^. The concept of hormesis suggests that low doses of toxic substances and radiation can cause modest biological responses that are opposite to those caused by higher doses of the same agents. This means that within the hormetic zone, the biological response to low exposures to toxins and other stressors is generally favorable^32^. There is also evidence supporting a link between protein aggregation and oxidative stress, which is involved in the development of various diseases^33^. Protein aggregation is the process by which unfolded or misfolded proteins expose their hydrophobic regions and form aggregates, which can ultimately lead to the endoplasmic reticulum (ER) stress and serious cellular damage^34^. The Unfolded Protein Response (UPR) is a cellular stress response related to ER stress. During the UPR, numerous genes are activated that sustain and protect the ER and improves the ER's capacity for protein folding^35^. Therefore, oxidative stress and the UPR are interconnected and studies have shown that hormesis can be induced by low levels of oxidative stress, which can stimulate the UPR and improve cellular health^36,37^.

To counter the oxidant stress after exposure to tolerated doses, the antioxidant defense system, which includes various cellular tools such as small antioxidant molecules, inducible antioxidant proteins, as well as UPR is activated^38^. Interestingly, different studies showed that inducing low doses of oxidative stress responses (in the range of oxidative eustress) positively affects recombinant protein production. In other words, oxidative stress and the ability to produce recombinant proteins are directly related. For instance, Delic et al. reported that constitutive co-expression of YAP1 (a transcription factor involved in the transcriptional response to oxidative stress) restores the cellular redox conditions of the protein-secreting *Pichia pastoris* by reoxidizing the cytosolic redox state to those of the wild type. This alteration led to increased levels of secreted recombinant protein. In another study, Martínez et al. constructed a yeast strain of *S. cerevisiae* (producing recombinant α-amylase) overexpressing the transcription factor Hap1, which activates several oxidative stress response genes. They demonstrated that Hap1 overexpression mitigates the negative effect of ROS accumulation and increases the production capacity of α-amylase during batch fermentations^39^. Simply put, although the accumulation of ROS negatively affects the production of recombinant protein, introducing low doses of oxidative stress led to the activation of various stress response pathways (and their interconnected pathways such as glycolysis, respiratory metabolism, cell membrane permeability, and etc.) that have favorable effects on the overall protein production, including the production of target recombinant protein^40,41^. Overall, low doses of stress can be beneficial in recombinant protein production, but it is important to manage stress levels to avoid negative impacts on host cell metabolism.

The production of recombinant protein by itself poses significant stress on the host cell; To meet the cell demand for more ATP in cells producing recombinant proteins, metabolic fluxes rewire toward oxidative phosphorylation and related pathways that are the main source of ROS in the cells. Besides, the recombinant protein synthesis represents a metabolic burden for host cells that implicates different kinds of stresses, including oxidative stress. This stress accumulation, due to the production of recombinant protein, may lead to the perturbation of redox balance toward the overwhelming stress that impairs cell functioning (distress), which negatively affects recombinant productivity and quality. Hence, oxidative stress-alleviating strategies (including the increase in oxidative stress-responsive genes dosage and expression) have been shown that could improve recombinant protein production in different hosts. For this purpose, cell engineering and environmental factors can be used as two main strategies^42,43^.

Furthermore, in the case of secretory recombinant protein, the use of strong promoters may result in the accumulation of proteins in the ER. As described previously, this accumulation may, in turn, lead to an overloaded unfolded protein that activates UPR stress response protection system^42^. According to recent findings, ROS and redox metabolism are closely associated with ER stress in different organisms, including yeasts^44^. So, synthetic overexpression of UPR regulators, such as HAC1, (using genetic engineering or environmental inducers) has been shown that raises recombinant protein production^45^.

In addition to the more conventional methods, such as genetic engineering or environmental optimization, to boost the efficiency of metabolic pathways, it has recently been noticed that some stressful conditions unexpectedly have a positive effect on heterologous protein production. Under stressful situations, the stress response genes would be up- or down-regulated transiently, shifting the microorganisms into a better state regarding protein folding, carbon or amino acids or lipid metabolism, and even membrane organization. On the other hand, activation of oxidative stress response genes alleviates the excessive ROS formation during recombinant protein production^46^. In this study, we attempted to provide a mildly stressful condition using non-lethal doses of CAP and evaluate its potential benefits in producing our recombinant target protein (eGFP) in yeast *Pichia pastoris*. Based on the outcomes of optical density and MTT assays, the CAP exposure times of 120, 180, and 240 s were selected and approved as non-lethal doses. Measuring fluorescent intensity (related to the amount of produced and secreted eGFP) indicated the positive effect of CAP exposure on the *Pichia pastoris* cells' productivity. The highest fluorescent intensity was recorded in cell culture media exposed for 240 s, an 84% improvement over the control. This increase was verified by the results of real-time PCR. However, the results of the Bradford assay indicated a 36% increase in the protein content of culture media after 240 s CAP exposure. Since eGFP has a high ROS quenching ability^47^, part of this fluorescent increase may be related to the effect of ROS on the eGFP structure that remains to be further studied.

Lin et al. figured out a list of genes related to the antioxidants and transcription factors that responded to oxidative stress caused by methanol. Then, they overexpressed each of these genes by a constitutive promoter (promoter of GAP) in *Pichia pastoris* and found that the ROS levels in these overexpressed strains were significantly lower than that of the control. Additionally, the expression level of the target protein (lipase) in overexpressed strains significantly increased. These results of Fig. 7a,b confirmed that the induction of antioxidant genes is very promising for improving recombinant protein production efficiency^48^. In the present study, we analyzed the expression of these genes five and 24 h after CAP exposure. All the selected genes showed a significant expression increase following CAP exposure. Furthermore, the pattern of expression changes was nearly the same for both hours. The durability of these expression modifications may be related to their applicability to long-lived species, such as H_2_O_2_.

The CAT1 with more than 2.5 fold increase showed the greatest change due to CAP exposure. SOD1, YAP1, ZWF1, SKN7, GLR1, and TRR1 are other genes that showed significant expression increases in both 5 and 24 h post-CAP exposure. However, the notable increase in AHP1 and GND2 gene expression after five h almost vanished after 24 h. These short-time increases may be due to a transient response to short-lived ROS. Although GSH2 gene expression showed a slight increase in expression, it was not statistically significant. The GSH2 increases the total glutathione cell content, which is one of the major ROS-scavenging antioxidants. This low effectiveness of GSH2 needs to be further studied by measuring other enzymes involved in glutathione metabolism or by directly examining the cell glutathione molecules content.

CAT1 and SOD are the main members of yeast's enzymatic oxidative stress defense system. SOD converts superoxide anion (O_2_^·−^) to H_2_O_2_ and O_2_, and CAT1 decomposites the H_2_O_2_ into H_2_O and O_2_ and protects the cells from the toxicity of H_2_O_2_ that can be lethal if not degraded^49^. Compared to the other ROS, H_2_O_2_ is the most stable and the least reactive ROS and can easily pass through the membrane, making it an appropriate signalling molecule. The effect of H_2_O_2_ is dose-dependent and exerts a diverse range of effects, from improving cell viability to cell death^50,51^. Here we found out that the CAP exposure produced a substantial concentration of H_2_O_2_ with rising trends over the time course of exposure. The lower levels of H_2_O_2_ produced in the YP medium (when compared to distilled water) demonstrated the potential ROS scavenging abilities of the YP culture medium that contains different amino acids, as proposed by Baik et al.^52^. Half of the generated H_2_O_2_ was still detectable after 24 h, confirming its stability in the cell culture medium. These persistent H_2_O_2_ molecules may account for the marked and long-lasting rise in CAT1 gene expression.

ZWF1, which sharply and persistently rises in response to CAP exposure, encodes for the enzyme catalyzing the initial stage of the pentose phosphate pathway and participates in oxidative stress adaptation. ZWF1 is one of the major enzymatic sources of NADPH, a crucial metabolic antioxidant^53^. The oxidant-sensitive transcription factors YAP1 and SKN1, the other oxidative stress response genes that were improved after CAP exposure, promote the transcription of genes that give the cell the capacity to combat oxidative stress^54,55^. Compared to the SKN1, YAP1 showed a higher and more stable increase after CAP exposure.

The OES results showed that the predominant species produced by this CAPJ were molecular nitrogen (N_2_) and its ionized derivative (N_2_^+^). Nitric oxide metabolites (NOx) are another signaling mediators involved in diverse physiological processes. In addition to the fast interaction of NO_x_ with the biological targets, it provides stable byproducts that continue the redox reaction chain. The measured concentration of NO_x_ was between 100 and 120 µM, which was similar to some previous works, such as Park et al., who studied the effect of CAP on human mesoderm-derived stem cells. They found that NO generated from CAP was the main factor for activating the expression of cytokines and growth factors^56^. NO is a signaling molecule that is widely conserved from bacteria to yeast, plant, and mammals. NO homeostasis is essential for the regulation of its physiological functions. Although little is known about its signaling, NO may play a role in yeast stress responses^57^. It is suggested that one aspect of NO physiology and pathophysiology is related to its interaction with thiol-containing proteins. Therefore, by reacting with the metal-binding thiol ligands in proteins, it may be able to control or disrupt yeast metal metabolism, and ions can impact cellular productivity and product quality^58,59^.

Stress responses may contribute to some of the observed effects of CAP exposure on the quantity of recombinant protein. However, some of the observed results may be explained by the impact of RONS on cellular constituents like proteins or lipids, as well as the oxidative impact of RONS on the structure and, subsequently, the function of recombinant proteins. In lab scale, using CAP may be considered a valuable strategy to improve recombinant protein production in a yeast host. Plasma jet arrays are the solution to larger scale treatment, they’re composed of parallel jets that provide the possibility of treating larger volumes and surfaces. Although this technique is not economically feasible on industrial scale, however, a detailed and extensive study of molecular modifications following CAP exposure could be inspiring for reverse metabolic engineering of host cells with improved potential in recombinant protein production.

## Materials and methods

### Strain, media, and cell culture condition

The recombinant *Pichia pastoris* yeast strain expressing recombinant eGFP was obtained from our previous study^60^. Briefly, the sequence of eGFP (GenBank:

AAF62891.1) was optimized according to the *Pichia pastors* codon bias and cloned in the pPink-αHC plasmid, in-frame with the α-MF signal peptide, and under the control of the AOX1 promoter. The recombinant plasmid was propagated in *Escherichia coli* cells and transformed in the PichiaPink™ expression system strain 4 (*ade2, pep2, pep4*). The yeast cells were grown in YPG medium (1% (w/v) yeast extract, 2% (w/v) peptone, 2% (v/v) glycerol) and induced for the recombinant protein production in YPM medium (same as the YPG medium except with 0.5% (v/v) methanol instead of the glycerol). The induction of expression with the AOX1 promoter was continued for three more days with a 1% methanol concentration per day. The culture was conducted at 250 rpm shaking and 28–30 °C.

### Cold atmospheric pressure plasma jet (CAPJ) and experimental setup

A DBD-based CAPJ by a ring-pin configuration was utilized. A quartz tube serves as the dielectric barricading sparks between the electrodes. The feeding gases were 99.999% pure helium (He) with three standard liters per minute flow rate. A sinusoidal AC power supply with a 20 kHz frequency triggered the gas discharge. Voltage was selected at 4 kV for a stable plasma plum.

After the growth of the *Pichia pastoris* yeast cells in the YPG medium, equal numbers of cells were inoculated to the 20 ml YPM medium. The CAP treatment was applied immediately at the beginning of the methanol induction phase, while the cell culture medium was stirred in a glass laboratory beaker. The jet was fixed at 1 cm height from the cell culture medium surface, and the CAP treatment with three different exposure times (120, 180, and 240 s) was examined. All cell culture media contained an equal number of cells, and all experiments were conducted in triplicate. The cell growth, viability, and produced recombinant proteins were assayed in the next three days of methanol induction.

**Figure 8:**
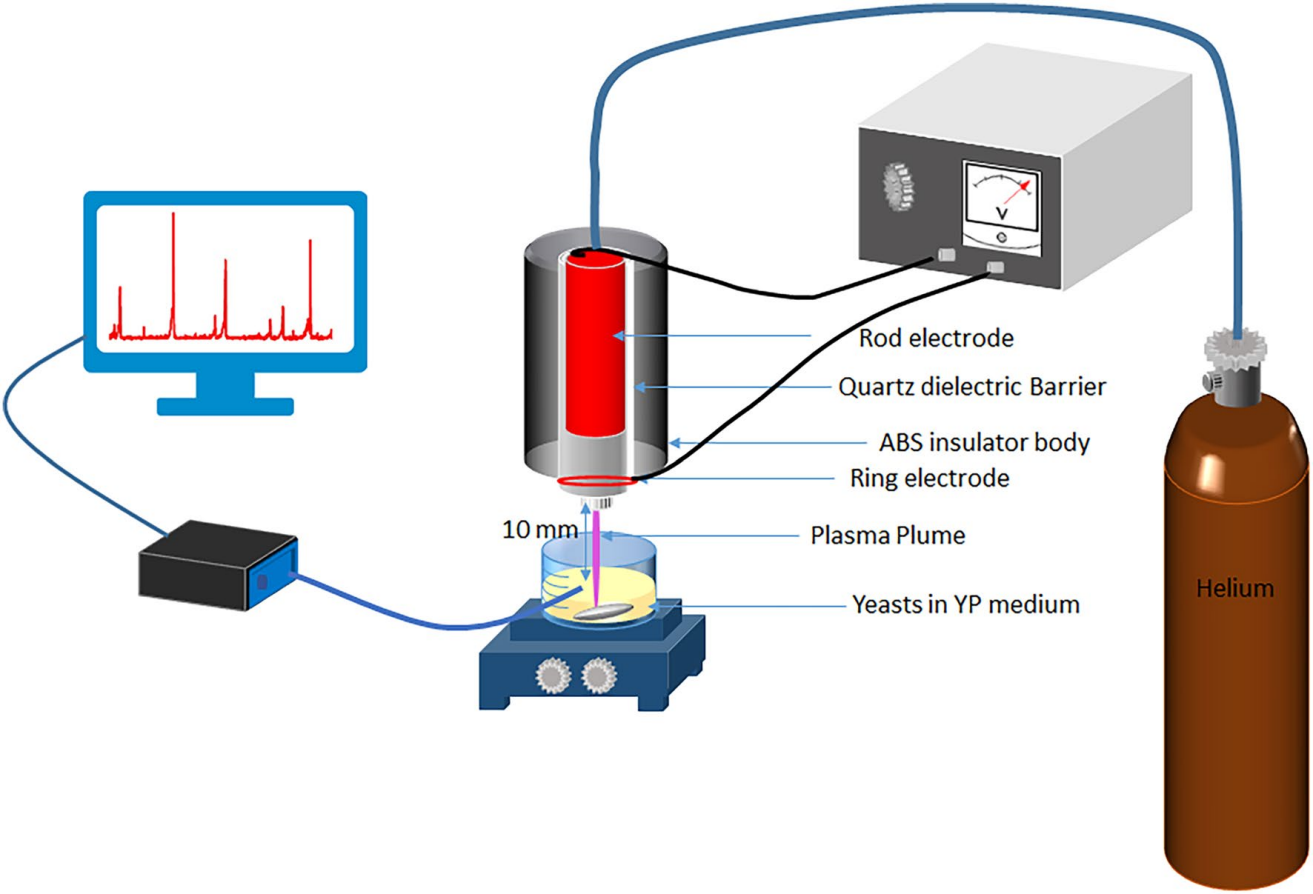
Plasma schematic and treatment setup.

The schematic representation of CAP treatment is shown in Fig. 8.

### Optical emission spectroscopy (OES) and plasma diagnostics

The plasma discharge characteristics and generated reactive species in the plasma plume were studied using OES. Two spectrometers with different resolution powers (Avaspec-3648 and -ULS 3648) were employed to record the optical emission and determine essential plasma species. They detect the emission at 200–1100 nm with 0.6 to 0.7 nm resolution and 280–440 nm with a 0.09 to 0.11 nm resolution, respectively. The recorded spectra were used to identify the reactive oxygen and nitrogen species (RONS) using the National Institute of Standards and Technology (NIST) spectra databases. The measured spectra were simulated using Specair software^61^ to estimate the rotational temperature of plasma, which is considered to be the gas temperature applied to the cell culture media. Technically, a temperature below 40 °C is classified as cold plasma.

### Cell growth and viability and protein quantification

OD_600_ of the culture medium was measured for three consecutive days to look into how CAP treatment affects yeast cell growth. Furthermore, the yeast cell viability after plasma treatment was assessed using -(4,5-dimethylthiazol-2-yl)-2,5-diphenyl tetrazolium bromide (MTT) assay, which is a simple colorimetric assay of cell survival. The test relies on the conversion of MTT into formazan crystal by living cells, which is an indicator of mitochondrial activity. The test was performed according to the previously described method^62^. Briefly, 30 μl of MTT (5 mg/ml; Bio-Idea, Iran) was added to 200 μl of cell suspension (containing 2 × 10^7^ cells) and incubated for three h at room temperature. Then, the solution was centrifuged at 10,000 rpm for two min, and the pelleted cells were washed with phosphate-buffered saline (PBS) and resolved in 70 μl DMSO. After 10 min incubation at room temperature, the content of the microtubes was transferred to a 96-well plate, and the absorbance was read at 490 nm using a Tecan infinite pro200 plate reader (Germany).

The protein concentration of cell culture media was measured using Bradford reagent (Navandsalamat, Iran) according to standard protocol^63^. The bovine serum albumin (BSA) was employed as the standard reference, and the data were normalized with a non-recombinant *Pichia pastoris* culture medium.

### Spectrofluorimetery

The fluorescence intensity of recombinant secreted eGFP and the impact of plasma treatment on the eGFP production/protein was measured by spectrofluorimeter (PerkinElmer LS 45). The excitation and emission wavelengths were selected at 450 and 507 nm, respectively. The isolated supernatants from untreated and plasma-treated clones expressing eGFP on the fourth day of induction were used for the test. The supernatant of non-recombinant *Pichia pastoris* and YP medium were considered negative controls.

### Effect of CAP on the cell culture pH and temperature

The temperature and pH of the cell culture medium were checked before and after (up to 10 min) of CAP treatment to ensure that there was no change in environmental parameters that would impact the observed results. A non-contact infrared digital camera (FLIR E4) and a pH-meter (Trans instruments bp3001, Japan) were used for measuring cell culture medium temperature and pH, respectively.

### RONS monitoring

Inducing RONS generation by CAP acts as the main mediator of CAP effects. As explained above, the RONS produced by plasma discharge was detected using OES. The generated species were measured immediately following and five and 24 h post-CAP treatment to measure the generated RONS in the CAP-exposed culture media and evaluate their stability. The H_2_O_2_ content of the culture medium was quantified using an colorimetric H_2_O_2_ assay kit according to the manufacturer's protocol (ZellBio GmbH, Germany). The kit includes a color reagent that reacts to produce a purple color proportional to the concentration of H_2_O_2_ in the sample. This dye is called xylenol orange and is present in an acidic solution with sorbitol and ammonium iron sulfate. The NO radical of the culture medium was measured using the Griess colorimetric kit according to its manual (P.K.A. Corp, Iran). This kit measures stable metabolic products of NO, i.e., nitrite and nitrate, due to the short lifetime of nitric oxide. The method relies on a two-step diazotization reaction, in which NO2 first reacts with sulphanilamide to produce a diazonium salt intermediate. This intermediate then reacts with N-1-naphthylethelene diamine to produce a chromophoric azo product that can be observed spectroscopically at 540 nm^64^. The total intracellular ROS was measured by staining using cell-permeant dye, 2',7'-dichlorodihydrofluorescein diacetate (H2DCFDA) (Teb Pazhouhan Razi, Iran). This dye is taken up by cells and in the presence of RONS is oxidized and converted to dichlorodihydrofluorescein (DCF), which has excitation and emission at 485 and 535 nm, respectively, giving it a green color. The fluorescence intensity was analyzed by BD FACSLyric™ flow cytometer (USA).

### Total RNA extraction, cDNA synthesis, and Real-time PCR

In the present study, we used non-lethal doses of plasma in yeast cell culture. However, the RONS molecules, the critical products of CAP exposure, operate as intracellular signaling molecules and would affect different aspects of cell functions, including gene expression. Here, we used real-time PCR to determine the effect of CAP exposure on the recombinant model protein at the mRNA expression level and evaluate the expression level of some genes previously verified to be associated with ROS response. The selected genes and their main function are listed in Table 1. The increasing trend of methanol-induced transcription factor (Mit-1) expression, which has a crucial role in methanol metabolism and was studied in this group's previous work, was checked to verify the obtained results^65^.

**Table 1:** The studied genes for evaluation of expression by real-time PCR.

Gene	Function
Catalase A (CTA1)	Decomposition of H_2_O_2_
Superoxide dismutase 1 (SOD1)	Dismutation of O_2_^-^
Suppressor of Kre Null (SKN7)	Transcription factor, control the expression of ROS-detoxifying
Yeast activator protein (YAP1)	Transcription factor, control the expression of ROS-detoxifying
Alkyl hydroperoxide reductase (AHP1)	Reduction of hydroperoxides
Glutathione reductase (GLR1)	Reduction of oxidized Glutathione
Thioredoxin reductase (TRR1)	A part of the Thioredoxin system
Glutathione synthetase (GSH2)	Synthesis of Glutathione, RONS scavenging
Zwischenferment (ZWF1)	Reduction of NADP^+^ to NADPH
6-phosphogluconatedehydrogenase (GND2)	Catalysis of NADPH regenerating
Methanol-induced transcription factor (MIT1)	Methanol metabolism

The expression analysis was performed before (as negative control), immediately after, and five and 24 h post cell culture CAP exposure. The yeast cell samples at the chosen times were frozen immediately at − 180 °C and kept at -80 °C until the test.

RNA extraction was performed using the total RNA Purification Kit (Jena Bioscience GmbH, Germany) according to the manufacturer's protocol with slight modifications. Briefly, the frozen cell samples were powdered by grinding in a pre-chilled mortar and occasionally adding liquid N_2_. After grounding to the fine powder, the lysis solution was added to the mortar. Lysed cells were homogenized using a syringe needle (G20) and incubated at room temperature for 30 min. The chloroform was used to promote phase separation and isopropanol to improve the RNA adsorption to the silica membrane. Bonded RNA molecules were washed and eluted by appropriate buffers supplemented by the kit. After measuring the isolated RNA concentration by Nanodrop (Thermo Fisher Scientific, USA) at 260 nm, the single-stranded cDNA was synthesized using RevertAid First Strand cDNA Synthesis Kit (Thermo Scientific, K1622, USA) according to the provided protocol. The synthesized cDNA was quantified at 280 nm using the Nanodrop instrument and used in real-time PCR analysis.

Real-time PCR was carried out using ExcelTaq™ 2X Fast Q-PCR Master Mix (SYBR, ROX) (SMOBIO, Taiwan)*,* and reactions were run on the Rotor-Gene Q 5plex HRM Platform (QIAGEN, Germany)*.* Successive reactions were repeated for 40 cycles of 95 °C for 15 s, 60 °C for 30 s, and the final extension at 60 °C for 30 s. The relative fold differences were calculated using the Pffafl method with Actin1 (ACT1) as the endogenous reference gene to normalize the results^66^*.* The used primers are depicted in Table 2.

**Table 2:** List of primers used in this study.

Primer name	Sequence (5′ 3′)	Amplicon size (bp)
eGFP	F : GCTGACCACTACCAACAGAACAC	143
	R : GCAGTAACAAACTCCAACAAGACC	
ACT1	F: CTCCAATGAACCCAAAGTCCAAC	100
	R: GACAAAACGGCCTGAATAGAAAC	
Mit1	F: GACGATCCAACCCCAAAGAATAC	103
	R : GTTGTTGTGGTGGGAAATATTGTTG	
CAT	F : TTCGACAACGCTAATCACGCTAAC	160
	R: TCACCTCAAACTCACCGAAAGCT	
SOD1	F : TCGAACAATCCTCCGAAAGCAGC	160
	R: CCGTGGGTCTTACCAAATGGGT	
AHP1	F: CTGGAGTCTTAGCTTGTGCTATTCC	147
	R: CGTCGATTTTCTCAATGAAAACAGG	
GLR1	F: GGTGCAAAGACCCTTTTGATTGAAG	168
	R: GTCCAGTTAAAGGAAAAATCGCC	
TRR1	F: GGGTATGTTGGCTAACGGTATCG	160
	R: GGTCTCGGTAATGATCTCAGTGCC	
GSH2	F: GCGATGTACCCCACCAATTTTGAG	162
	R: GCCCAGCCATTCCTGATTTTTGA	
ZWF1	F: AGGGCGACGAGGACAAAGTTC	164
	R: GGGCAAAGCTAAGTAGAACAACCTG	
GND2	F: GGTTTCACCGTCGTCGCTTACA	166
	R: CGGGATTACCAGCCTTGACCAATA	
YAP1	F: ACTGCCGACAAGTATACCGC	190
	R: CAATGGGCTGTTGCTTCCAC	
SKN7	F: CCACATCAACTGCCGAAAGG	181
	R: GCGCCTGTCGAGACTAATG	

## Data Availability

Data underlying the results presented in this study are available from the corresponding author upon reseanable request.
